# Spectroscopic insights into the mechanism of anammox hydrazine synthase

**DOI:** 10.1016/j.jbc.2025.110771

**Published:** 2025-09-29

**Authors:** Wouter Versantvoort, Rainer Hienerwadel, Christina Ferousi, Pieter van der Velden, Catherine Berthomieu, Laura van Niftrik, Frauke Baymann

**Affiliations:** 1 Microbiology, RIBES, Faculty of Science, Radboud University, Nijmegen, The Netherlands; 2 LGBP, BIAM, Aix-Marseille University, Marseille, France; 3 IPM, BIAM, CEA-Cadarache, Saint-Paul-lez-Durance, France; 4 Laboratoire de Bioénergétique et Ingénierie des Protéines, CNRS/AMU, Marseille, France

**Keywords:** anammox, hydrazine synthase, heme, EPR spectroscopy, FTIR spectroscopy, electrochemistry

## Abstract

Anaerobic ammonium oxidizing (anammox) bacteria oxidize ammonium with nitrite as electron acceptor. Hydrazine is a free intermediate in this metabolism, produced by the enzyme hydrazine synthase. Hydrazine synthase is a tetraheme cytochrome *c*, containing two proposed active site hemes (γI and αI), connected by an intra-enzymatic tunnel. These structural features resulted in an initial hypothesis of its reaction mechanism: nitric oxide is reduced to hydroxylamine which is condensed with ammonium to form hydrazine. Here, investigations by electrochemically induced optical and infrared difference spectroscopy and electron paramagnetic resonance revealed two low potential low spin hemes, αII and γII, with midpoint potentials of ∼ -330 mV *(versus* SHE). Heme γI showed redox transitions in the range of 0 mV, featuring both low-spin and high-spin characteristics possibly due to implication of an aspartic acid, connected to heme γI axial site by a OH^-^/H_2_O. Furthermore, electron paramagnetic resonance spectroscopy confirmed the ability of heme γI to bind NO in the reduced state. Heme αI exhibited a rhombic high spin signal, in line with its ligation by a proximal tyrosine observed in the crystal structure. Neither dithionite nor potentials of −610 mV reduced this heme, indicating a very low midpoint potential. *In vivo* chemistry at this heme αI, the candidate for the comproportionation of hydroxylamine and ammonium, is thus likely to be initiated solely on the oxidized heme, in contrast to previously reported DFT calculations.

Anaerobic ammonium oxidizing (anammox) bacteria are chemolithoautotrophic microorganisms that make a living by converting ammonium and nitrite to dinitrogen gas, with hydrazine as a unique free intermediate (1, 2, 3). Anammox bacteria are ubiquitous in both natural and engineered ecosystems, where they contribute significantly to the release of fixed nitrogen from their environment. It is estimated that approximately 50% of the nitrogen loss from the ocean is due to anammox activity (4, 5). Furthermore, they are successfully applied in over a 100 wastewater treatment plants worldwide (6, 7, 8).

The catabolic reactions of anammox metabolism take place in a dedicated intracellular compartment termed the anammoxosome (1, 2, 9). The current working hypothesis is that nitrite is initially reduced to nitric oxide by nitrite reductase (2, 10). This nitric oxide is subsequently further reduced and condensed with ammonium to form hydrazine by hydrazine synthase (HZS) (3). Hydrazine is then oxidized to dinitrogen gas by hydrazine dehydrogenase, releasing four low potential electrons (11). These electrons, in a yet to be discovered manner, must be shuttled through a membrane-bound respiratory chain contributing to the maintenance of the proton motive force required for ATP synthesis, before they return into the anammoxosome to resume the first two steps of the anammox reaction cycle (2, 12).

The formation of hydrazine as a free metabolic intermediate is unique to anammox bacteria as is HZS, the enzyme responsible for its production. The purification and crystallization of HZS directly from native *Kuenenia stuttgartiensis,* the type strain for anammox bacteria, allowed for an initial hypothesis of a two-step reaction mechanism. HZS crystallized as a dimer of the HZSα, HZSβ, and HZSγ heterotrimer (Fig. 1), with four covalently attached heme *c* groups per heterotrimer. The γ-subunit contains two *c*-type cytochromes and shows homology to the family of MauG and bacterial cytochrome *c* peroxidases. Bis-His ligated heme γII (Figs. 1 and 2*B*) is surface exposed and proposed to accept electrons from a cytochrome *c* partner (13), which it shuttles to heme γI (Figs. 1 and 2*A*). Heme γI is covalently bound by three cysteines and is coordinated by a histidine and a hydroxide/water. This heme is proposed to catalyze the three-electron reduction of nitric oxide to hydroxylamine (14). Hydroxylamine then diffuses through an intra-enzymatic tunnel to the second active site heme αI (Figs. 1 and 2*C*). Ammonium diffuses through a second, minor tunnel to heme αI where it is condensed with hydroxylamine to from hydrazine. Strikingly, the histidine (His587) from the CxxCH binding motif of heme αI is coordinating a zinc residue and the heme has a tyrosine (Tyr591) as proximal ligand, reminiscent of catalases. Its protein environment, however, is not homologous to any other known structure. The α-subunit contains an additional bis-His ligated *c-*type heme αII (Figs. 1&2D), for which no function is yet hypothesized as it is not within efficient electron transfer distance from other HZS hemes. This heme is embedded in a typical type I cytochrome *c* fold. The β-subunit consists of a β-propeller structure, but does not contain any cofactors. It likely fulfills a structural role and contains a loop region near the intra-enzymatic tunnel, which might regulate access to the α-subunit (14).

(1) $$NO+3\;e^{-}+3\;H^{+}\;\to \;NH_{2}OH\;E_{0}’=-30\;\text{mV}$$

(2) $$NH_{2}OH+NH_{3}\;\to \;N_{2}H_{4}+H_{2}O$$

(3) $$NO+NH_{3}+3\;e^{-}+3\;H^{+}\;\to \;N_{2}H_{4}+H_{2}O$$

**Figure 1:**
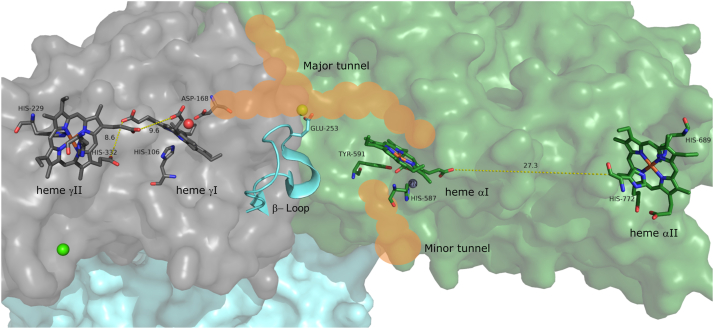
**Crystal structure of the HZS dimer from *Kuenenia stuttgartiensis*, containing four *c*-type hemes per αβγ-monomer**. The γ-subunit (in *gray*) consists of a bis-His ligated heme yII, which is surface exposed near a cytochrome *c* binding site. Within 10 Å sits heme yI, coordinated by a proximal histidine and a hydroxide. The α-subunit (in *green*) contains a surface exposed bis-His coordinated heme αII and a 5-coordinated heme αI, with a tyrosine as proximal ligand. Heme αI and heme γI are connected by an intra-enzymatic tunnel (in *orange*). A loop of the β-subunit (in *blue*) (245–260) approaches the tunnel and could regulate diffusion of molecules between the two hemes (PDB:5C2V) (14). PDB, Protein Data Bank; HZS, hydrazine synthase.

Hydrazine synthesis is at the heart of the anammox metabolism and unique among biological energy conversion reactions. The molecular function of the enzyme proposed to catalyze this reaction is based on structural data and basic chemical knowledge. Experimental data to confirm the proposed mechanism (or come up with a different one) are missing. The redox properties of the enzyme cofactors and the dynamics of the protein at work can be assessed by electrochemistry coupled to various spectroscopic methods. Here, we performed the entire purification procedure under anaerobic conditions to preserve the native properties of the enzyme. We then combined electrochemically induced FTIR difference spectroscopy, electron paramagnetic resonance (EPR) and optical spectroscopy to study the molecular mechanism of HZS and compared the data to the proposed reaction mechanism.

## Results and discussion

### UV-visible spectroscopy

Purified *K. stuttgartiensis* HZS exhibited a ferric heme *c* UV-visible spectrum in the “as isolated” state (Fig. 3*A*, black line) with characteristics for five- and six-ligated hemes as a Soret band maximum at 405.5 nm, a broad feature in the Q-band region between 500 and 600 nm, and a charge transfer band around 620 nm. Upon addition of dithionite, two distinct spectra appeared in a time-dependent manner. Immediately after dithionite addition (dith red 1), part of the Soret band shifted to 419 nm with a concomitant appearance of a split alpha band with maxima at 548.5 nm and 554.5 nm (Fig. 3*A*, red line). No intensity decrease in the charge transfer band was detected. After incubating with dithionite for 5 min (dith red 2), the contribution at 405.5 nm was further decreased and the Soret maximum was shifted to 420 nm. This Soret band contained two shoulders at 398.5 and 430 nm, respectively. Furthermore, an alpha band maximum typical for a reduced low spin heme was observed at 553.5 nm, overlaying the split alpha band (Fig. 3*A*, blue line). In addition, the charge transfer band at 620 nm drastically decreased in intensity, which together with the appearance of the 430 nm shoulder indicated the reduction of a high spin heme. The reduced minus oxidized difference spectra (Fig. 3*B*) calculated for the immediate reduction step, *i.e.* “dith red 1” minus ”as isolated” (Fig. 3*B*, red line) and for the second reduction step, *i.e.* “dith red 2” minus “dith red 1” (Fig. 3*B*, blue line), corroborated the appearance of three distinct spectral components. The “dith red 1” minus “as isolated” spectrum showed a shift of the Soret band from 403.5 nm to 420 nm and the appearance of the split alpha band with maxima at 548 and 554.5 nm. The difference spectrum of the 5 min incubated “dith red 2” sample minus “dith red 1” showed a further decrease of the Soret at 403 nm with an increase of absorbance at 421 nm and a narrow alpha band at 553 nm. In addition, a clear shoulder appeared at 430 nm and the charge transfer band at 620 nm disappeared.

**Figure 2:**
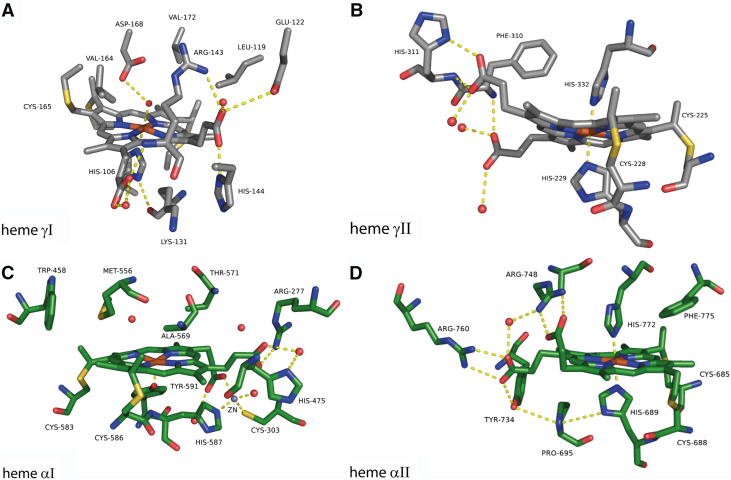
**Close-up view of the four hemes of *Kueneniastuttgartiensis* HZS and their immediate environment**. (PDB:5C2V) (14). *A*: γI, *B*: γII, *C*: αI, and *D*: αII. PDB, Protein Data Bank; HZS, hydrazine synthase.

**Figure 3:**
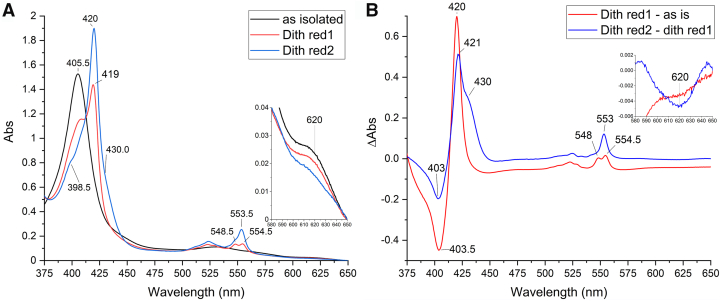
**UV-visible spectra of *Kuenenia stuttgartiensis* HZS***A*, “as isolated” (*black*) and reduced with dithionite. Immediately after dithionite addition (dith red1, *red*), part of the hemes got reduced, resulting in the appearance of two alpha bands with maxima at 548.5 and 554.5 nm and a shift in the Soret region from 405.5 nm to 419 nm. After incubating with dithionite for 5 min (dith red 2, *blue*), the alpha region showed a peak with a maximum at 553.5 and the Soret peak was shifted to 420 nm and a shoulder at 398.5 nm became visible. The inset shows the presence of a charge transfer band at 620 nm, typical for high spin hemes. This band did not decrease immediately upon dithionite addition (*red*), but decreased after 5 min incubation of the sample with dithionite (*blue*). *B*, the difference spectrum of HZS immediately after dithionite addition minus the “as isolated” spectrum (*red*), showed a shift in the Soret maximum from 403.5 to 420 nm and the appearance of the double peak in the alpha region with maxima at 548 and 554.5 nm. The difference spectrum of HZS incubated for 5 min with dithionite minus the initial dithionite reduced spectrum (*blue*) showed the appearance of a peak in the Soret region at 421 nm with a shoulder at 430 nm and a peak at 553 nm. The inset shows a zoom of the 580 to 650 nm range, where the decrease in the intensity of the charge transfer band at 620 nm was visible. HZS, hydrazine synthase.

**Figure 4:**
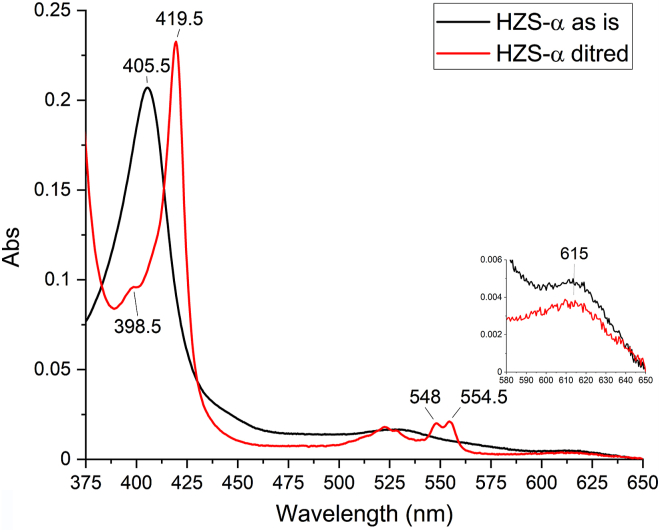
**UV-visible spectra of the isolated HZS α-subunit from *Kuenenia stuttgartiensis* in the “as isolated” (*black*) and dithionite reduced (*red*) state**. Upon dithionite reduction, two peaks appeared in the alpha region with maxima at 548 and 554.5 nm and the Soret maximum shifted to 419.5 nm. A minor peak at 398.5 nm became apparent in the dithionite reduced sample. The inset shows a zoom of the 580 to 650 nm region, where the presence of a charge transfer band at 615 nm was detected, which remained upon dithionite addition. HZS, hydrazine synthase.

The UV-visible spectra of the purified HZS α-subunit showed a similar “as isolated” spectrum (Fig. 4, black line) as the complete HZS, with a Soret maximum at 405.5 nm, a broad shoulder in the Q-band region and a charge transfer band at 615 nm. Reduction of the α-subunit with dithionite resulted in a shift of the Soret band to 419.5 nm, with a shoulder at 398.5 nm, and the appearance of a split alpha band with maxima at 548 and 554.5 nm (Fig. 4, red line). This spectrum was very similar to that obtained immediately after dithionite addition to the whole enzyme (Fig. 3*A*, red line) and indicated that these contributions stem from hemes in the α-subunit. Additional incubation of the α-subunit with dithionite did not result in the appearance of a shoulder at 430 nm nor an alpha band at 553 nm nor the disappearance of a charge transfer band, as was observed for the whole enzyme, which indicated these later contributions stem from hemes of the γ-subunit, and that a high-spin state heme remained oxidized in the presence of dithionite in the α-subunit.

### EPR spectroscopy

**Figure 5:**
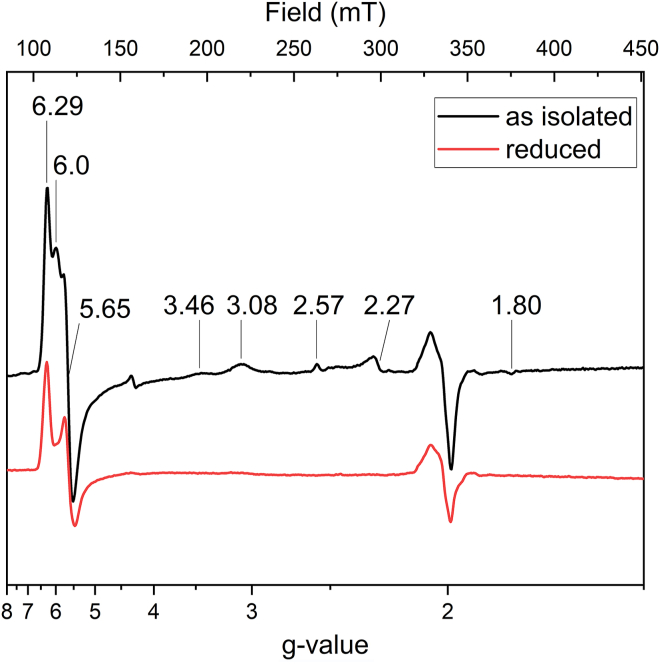
**X-band EPR spectra of *Kuenenia stuttgartiensis* HZS recorded at 25 K with a modulation amplitude of 6.5 mT.** As isolated (*black*) anaerobic HZS showed three signals in the high-spin region with g-values of 6.29, 6, and 5.65, two highly anisotropic low spin signals with g-values of 3.46 and 3.08, and one low-spin signal with g-values of 2.57, 2.27, and 1.80. Finally, a radical signal at g = 2 was observed. Reduction of HZS with one equivalent of dithionite (*red*) resulted in the disappearance of all signals except for part of the high spin signals with g-values of 6.29 and 5.65 and the signal around g = 2. HZS, hydrazine synthase; EPR, electron paramagnetic resonance.

The EPR spectrum of the “as isolated” anaerobically purified HZS showed signals reminiscent of the previously published aerobically purified enzyme (14), with some noticeable differences. A slightly rhombical distorted signal (HSp1) with a g_z_ at 6.29 and g_y_ at 5.65, and an axial signal (HSp2) at g = 6 in the high spin region (Fig. 5, black line) were observed in both preparations. The g_x_ signal, expected at g = 2, cannot be observed since radical features dominate this spectral region (see below).

**Figure 6:**
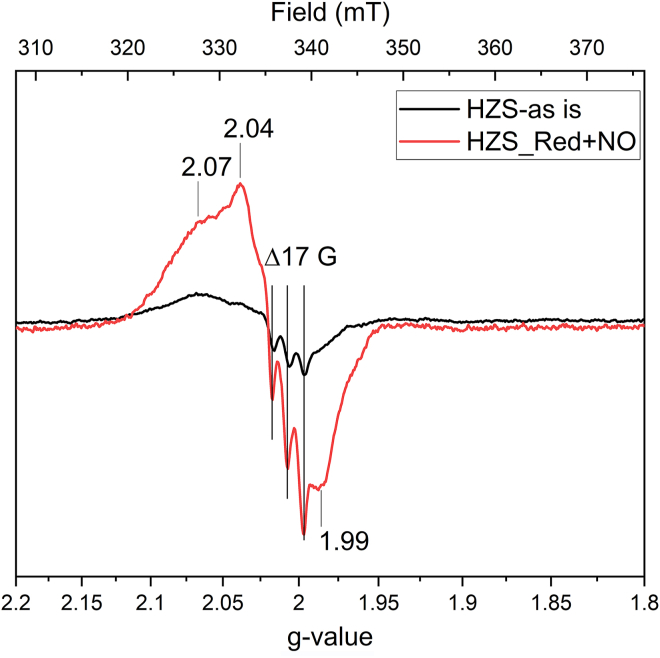
**X-band EPR spectra of *Kuenenia stuttgartiensis* HZS recorded at 60 K with a modulation amplitude of 6.5 mT**. “As isolated” HZS (*black*) and a dithionite reduced HZS treated with ∼95 μM NO (*red*); both showed a radical signal with a 16 G hyperfine split of the g_y_-tensor. Spectra were normalized on protein concentration. HZS, hydrazine synthase; EPR, electron paramagnetic resonance.

**Figure 7:**
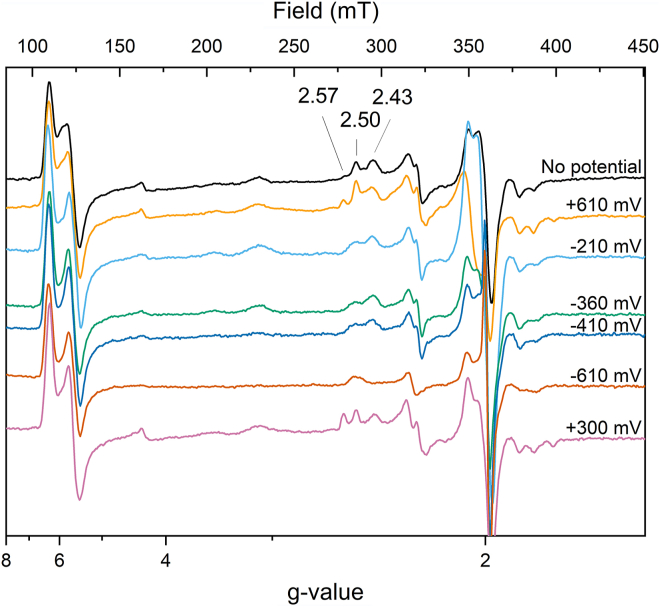
**X-band EPR spectra of *Kuenenia stuttgartiensis* HZS recorded at 25 K with a modulation amplitude of 6.5 mT**. Samples at pH 7 were poised to different potentials on a carbon felt electrode in the following order: *black*: sample as prepared, *orange* +610 mV, *cyan* -210 mV, *dark green*: -360 mV, *blue* -410 mV, *red* -610 mV, and *purple* +300 mV. HZS, hydrazine synthase; EPR, electron paramagnetic resonance.

The previously published, unexplained signals at g = 7.5 and 6.8 (14) were not observed with the anaerobically purified enzyme, but could be induced by exposure of the enzyme to O_2_ for 1 h at room temperature (data not shown). Two highly anisotropic low-spin signals, HALS1 with g_z_ at 3.08 and HALS2 with g_z_ 3.46, respectively, were consistently observed for the previous aerobic and the here reported anaerobic purified enzyme. Due to the different angles between the histidines ligating the low-spin hemes, 30° for heme αII (Figs. 2*D*) and 40° for heme γII (Fig. 2*B*), respectively, HALS1 were attributed to heme αII and HALS2 to heme γII (14, 15, 16). The deviation between the observed g-value and the value expected from the angle between the ligand planes could be attributed to other factors influencing the position of the g-value such as distortion of the heme plane. In addition, up to three low-spin ferric heme signal (LS) were observed with g_z,y,x_-values at 2.44, 2.23, 1.9, at 2.50, 2.27, 1.86 and at 2.57, 2.21, 1.80. The respective amplitude of their contributions depended on preparation and was influenced by the presence of mediators in the sample (compare Figs. 5 and 7). Integration of the ensemble of these signals revealed that they represent 20 to 30% of one heme. The aerobically purified enzyme showed an intense LS signal at g_z,y,x_ = 2.67, 2.3, 1.73 that was never observed in an anaerobically purified enzyme (14), not even after exposure of the anaerobically purified enzyme to oxygen. The different LS signals titrated over a broad range (Fig. 7) in the electrochemically induced redox titration followed by EPR spectroscopy and did not completely vanish when a potential of −610 mV was applied to the sample. Dithionite addition, however, reduced all these hemes (Fig. 5). We attribute this to an incomplete equilibration of the sample and to an enhanced variability of the signals due to the presence of mediators. Interestingly, reoxidation of the sample with one equivalent of ferricyanide after reduction by one equivalent of dithionite or by electrochemistry on a carbon electrode by applying a potential of +300 mV after reduction at −610 mV (Fig. 7) recovered all signals. Addition of ferricyanide to an untreated sample did not induce increase in signal amplitude or appearance of additional signals, indicating that the enzyme was fully oxidized after purification.

To better resolve the features in the radical signal observed at g ≈ 2, spectra were recorded at 60 K on a narrow field range with a decreased modulation amplitude of 6 G. These conditions resolved the spectral characteristics of an NO radical bound to a reduced heme. The three lines resulting from interaction of the radical spin with the nuclear spin of the nitrogen were spaced by 48 MHz (17 G), without further hyperfine splitting, which is characteristic for NO bound as a fifth ligand to a heme Fe(II) (Fig. 6, black line) (17). Quantification of the signal showed it amounts to 30 to 35% of one heme. As NO is one of the substrates for HZS, part of the enzyme might have been trapped in a NO-bound state upon cell disruption. Incubating reduced HZS with NO resulted in an increased signal in this spectral region (Fig. 6, red line) and we estimated the spin concentration to about two-thirds of HZS present in the sample (28 μM NO-bound heme for 45 μM HZS). Interestingly, an increased NO signal was also observed when poising the sample to −210 mV without extra NO addition, indicating that some NO was copurified with HZS and revealed upon reduction of heme γI (Fig. 7, red line). This signal disappeared when the potential was further lowered. Exposure of the anaerobically isolated HZS to oxygen resulted in the disappearance of the NO signal as well (data not shown).

Reduction of HZS with one equivalent of dithionite resulted in the disappearance of all low-spin signals and the axial high-spin signal, whereas the rhombic high-spin signal remained (Fig. 5). Further addition of dithionite did not change this neither did electrochemical reduction to −610 mV (Fig. 7). In the optical spectra (Fig. 3*A*, blue line) the presence of an oxidized high-spin heme is reflected by the signal at 398.5 nm and the persistence of part of the charge transfer band at 615 nm upon dithionite reduction. Both features are visible and even more pronounced in the optical spectra of the isolated alpha subunit after reduction of the low-spin heme αII by dithionite (Fig. 4, red line). We therefore attribute the rhombic EPR signal with at g_z_ 6.29 and g_y_ at 5.65 to the Tyr-ligated heme αI. The reducible, axial high-spin heme signal HSp2 then can be attributed to the histidine-ligated heme γI (Fig. 2*A*). Its small signal intensity, when compared to the rhombic signal of heme αI showed that only part of the population of the γI heme is in a spin state that gave rise to signal in the g = 6 region. Another population of the same heme may be at the origin of the LS signals and the NO-bound signal around g = 2. Taking the quantification of the two latter signals into consideration, the axial high spin signal would account for 30 to 40% of the population of one heme. The interconversion between HSp2 and LS signals is further corroborated by the fact that upon reoxidation of the reduced sample, EPR spectra revealed variable amplitudes for HSp2 and LS with, notably, a decrease in the amplitude of the HS species and a concomitant increase in the low-spin heme signal intensity at g_z_ = 2.57, g_y_ = 2.27 g_x_ = 1.8. Finally, the individual potentials of the two HALS signals, HALS1 and HALS2, could not be resolved in this experiment.

In summary, the four different HZS hemes all have distinct EPR signatures. The two highly anisotropic signals could be attributed to hemes αII and γII, based on the angle between their His ligands observed in the structure. Clear signals in optical spectroscopy make those hemes accessible for electrochemical studies followed by UV/visible spectroscopy and their reversible redox behavior allows for redox-induced FTIR difference spectra. Heme αI, however, showed a distinct rhombic high-spin signal, which was irreducible by all tested procedures, prohibiting further investigation of this heme with electrochemical methods that monitor redox-induced spectral changes. Finally, EPR spectroscopy revealed a mixed HS/LS state as well as an NO-bound state ascribed to heme γI. Here, EPR allowed to detect one high-spin and several low-spin components the proportions of which varied.

### Optical redox titration

**Figure 8:**
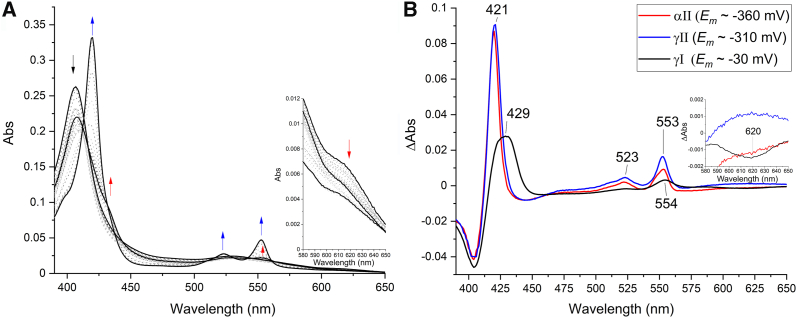
**UV-visible spectra of *Kuenenia stuttgartiensis* HZS during a reduction/oxidation cycle in a redox titration***A*, first reduction and subsequent oxidation at pH 8. Upon reduction, the Soret band at 405.5 nm and the charge transfer band at 620 nm decreased with a concomitant initial increase of a Soret band at 430 nm and an alpha-band at 554 nm (*red arrows*). Below -200 mV a Soret band with a final maximum of 419.5 nm started appearing in combination with the appearance of Q-bands at 523 and 553 nm, without further changes to the charge transfer band (*blue arrows*). *B*, UV-visible spectra of the first reduction cycle of a redox titration of *Kuenenia stuttgartiensis* HZS at pH 8 as extracted from the titration data by a global fit analysis (QSoas (47)) with three components. HZS, hydrazine synthase.

**Figure 9:**
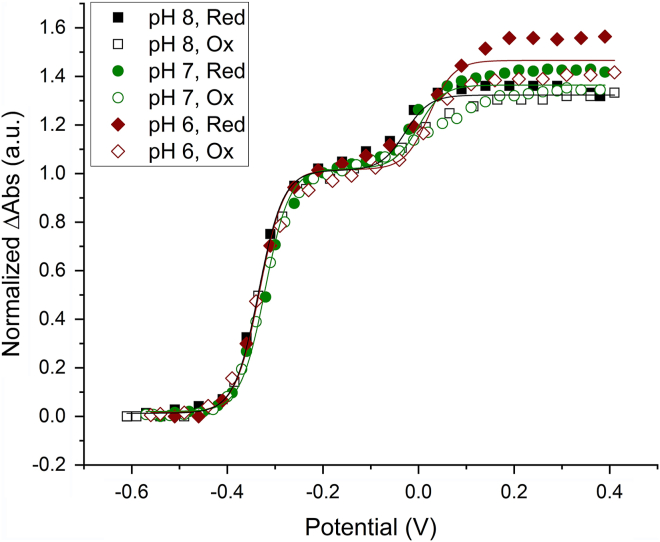
**Normalized amplitude changes of the absorbance difference between 406 nm and 412 nm as a function of the reduction potential of HZS at pH 6 (*red*, *diamond*), pH 7 (*green*, *circle*), and pH 8 (*black*, *square*).** During the oxidation (*open symbols*) and reduction (*closed symbols*), absorbance changes followed the same behavior in the low potential range, but not in the high potential range, where they deviated from an n = 1 Nernst curve. Furthermore, hysteresis between reduction and oxidation was observed at the high potential range. The lines represent the best fit to the combined reduction and oxidation data, which is a sum of two n = 1 Nernst curves with redox midpoint potentials of −330 and 20 mV for pH 6, -320 and 0 mV for pH 7, and -330 and −30 mV for pH 8, respectively. Data were normalized on the amplitude of the low potential wave to allow for comparison between different pH values, for which sample concentration varied. HZS, hydrazine synthase.

Optical redox titrations of HZS at pH 8 (Fig. 8*A*), pH 7 and pH 6 were used to determine the reduction potentials of the different *c*-type hemes. At the beginning of the reduction, the Soret band at 405.5 nm decreased and an initial absorbance increase at 430 nm was observed in combination with an alpha band at 554 nm and a decrease of the charge transfer band at 620 nm. Below −200 mV, a Soret band with final maximum at 419.5 nm started appearing in combination with Q-bands at 523 and 553 nm. To obtain the individual heme reduction potentials, absorbance changes at 406 minus 412 nm (for the maxima of the Soret bands in the oxidized and reduced state, respectively) were plotted against the applied redox potential (Fig. 9). Two separate absorbance decreases indicated heme reduction occurring around 0 mV and −330 mV. The low potential wave was fully reversible between reductive and oxidative directions and did not change significantly as a function of pH in the range investigated. Redox midpoint potentials of −320 mV for pH 7 and -330 mV for pH 6 and 8 were found. The corresponding spectra showed a Soret band at 420 nm and an alpha band at 553 nm. Using a global Nernst fit to analyze the total spectroscopic data set allowed us to extract two slightly different reduced minus oxidized difference spectra from the low potential wave, with reduction potentials of −310 and −360 mV (Fig. 8*B*, blue and red line). The spectrum corresponding to the lowest redox range showed an asymmetric alpha band (Fig. 8*B* red line), reminiscent of the split-peak spectrum seen for heme αII in the isolated alpha subunit. This heme may therefore have a slightly lower potential than heme γII. Depending on preparation, the difference between the two low-spin heme spectra was more or less pronounced.

**Figure 10:**
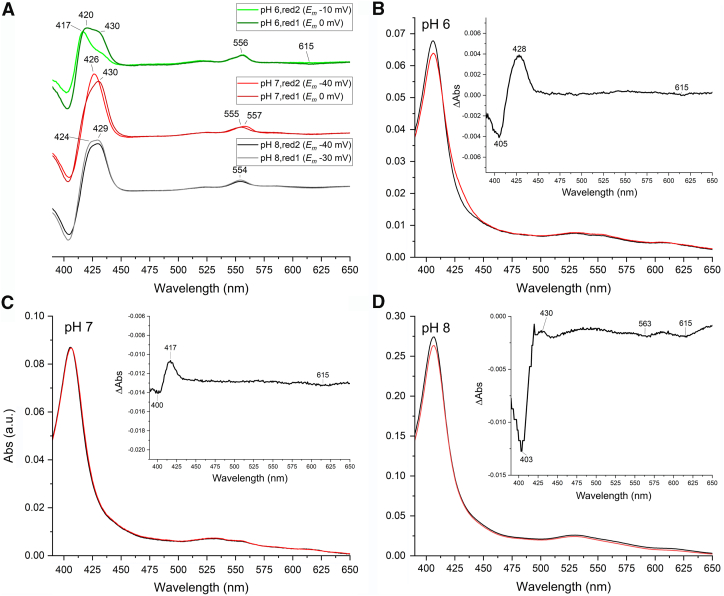
**Visible spectra of HZS at different pH-values.***A*, reduced minus oxidized difference spectra for the high potential component of *Kuenenia stuttgartiensis* HZS at pH 6 (*green*), pH 7 (*red*), and pH 8 (*black*) during the first reduction cycle (*light hue*) and second reduction cycle (*dark hue*). Each spectrum is normalized on the amplitude of the low potential wave. Absolute full initial spectrum (*black*) and first reoxidized spectrum (*red*) at pH 6 (*B*), pH 7 (*C*), and pH 8 (*D*). Spectra were normalized on the Soret maximum of the initial spectrum. Insets show the difference spectrum of the reoxidized minus initial spectrum at the respective pH values.

The high potential range showed a higher variability between different experiments and as a function of pH with +20 mV, 0 mV and −30 mV for pH 6, 7, and 8, respectively. The corresponding reduced minus oxidized difference spectrum had a broad Soret maximum at 429 nm and a weak alpha band at 554 nm, in addition to a charge transfer band at 620 nm. This indicated a mixed high spin/low spin contribution to this spectrum, which was in line with the g = 6 and the g_z,y,x_ = 2.57, 2.27, 1.8 EPR signals, attributed to heme yI. Upon successive redox cycles the amplitude and spectral characteristics associated with this redox transition evolved in a pH dependent manner (Fig. 10*A*). During the first reduction, a mixture of high-spin and low-spin heme is observed for all three pH values. At pH 6, the relative contribution of the low-spin signal is higher compared to pH 7 and 8 as evidenced by a broad feature with maxima at 420 and 430 nm for low and high spin reduced hemes. On the second reduction, the high potential difference spectrum at pH 8 is unchanged compared to the first reduction, whereas for pH 6 and 7 there are considerable changes. Analysis of the raw spectra showed that at pH 8 there is only some overall sample loss occurring (Fig. 10*D*). At pH 6 (Fig. 10*B*), after the first reduction the high-spin signal with a Soret band at 430 nm remained almost completely reduced even when oxidizing potentials were applied. At pH 7 (Fig. 10*C*), HZS was nearly completely reoxidized. Only a small increase in the Soret band at 417 nm and a decrease in signal amplitude at 615 nm in the reoxidized spectrum compared to the initial spectrum were observed. Combined with the change of the Soret band observed between the first and second reduction (Fig. 10*A*, green) it indicated a difference in contribution of high- and low-spin components. This was in agreement with the observation made by EPR at pH 7, where the axial g = 6 signal attributed to the high-spin population diminished upon reoxidation. Whereas, with the EPR data alone we would not have been able to decide whether part of the high-spin heme population was lost or stayed reduced, the combination of EPR and visible data clearly indicated that part of the population stayed reduced and part of it changed its spin state. This effect is most pronounced at pH 6 (Fig. 10).

### FTIR difference spectroscopy

With the redox midpoint potentials determined by optical spectroscopy at hand, the molecular changes associated with the reduction of the hemes were investigated using electrochemically induced FTIR difference spectroscopy. Heme modes as well as contributions from axial ligands, propionate groups, amino acid side chains, or the protein backbone are expected to contribute to the FTIR difference spectra. Reduced-minus-oxidized FTIR difference spectra were recorded in the potential ranges previously determined by optical spectroscopy to separate the redox transitions of the individual hemes.

**Figure 11:**
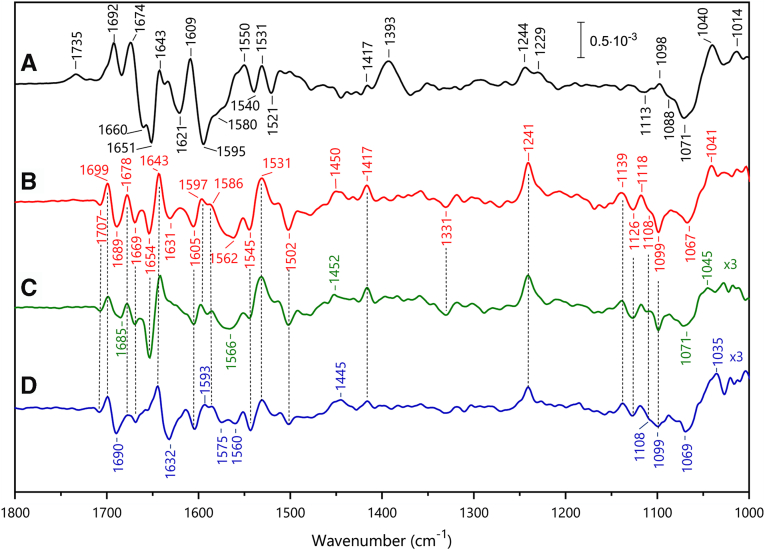
**Electrochemically induced FTIR difference spectra of *Kuenenia stuttgartiensis* HZS at pH 8.***A***,** -210 mV minus +390 mV, *B*, -560 mV minus -210 mV , *C*, -360 minus -210 mV, and *D*, -610 mV minus -410 mV. Spectra of C and D are enlarged three times compared to spectrum B. HZS, hydrazine synthase.

The spectrum recorded in the −210 to +390 mV range (Fig. 11*A*) corresponds mainly to the reduction of the high potential heme γI, which undergoes redox reaction around 0 mV (see above). This spectrum showed a positive band at 1735 cm^−1^, in a spectral range where only protonated carboxylic (COOH) groups are expected to contribute. This positive band (*i.e.*, without negative counterpart above 1690 cm^-1^) indicated that heme γI reduction was accompanied by the protonation of a carboxylate group. A negative band at 1580 cm^-1^ was in line with the contribution from a corresponding deprotonated carboxylate group (COO^-^) in the oxidized state (18). In addition, broad bands, negative at 1071 cm^-1^ and positive at 1040 cm^-1^ were assigned to proton release by the Tris buffer upon heme reduction (19). This suggested that reduction of heme γI was accompanied by a proton uptake from the buffer to a carboxylate group.

Protonation of a propionic group from a heme or a carboxylic group from an Asp or Glu side chain may cause this signal. The ν(C=O) mode of protonated propionic groups is expected to contribute at 1700–1665 cm^−1^, *i.e.*, at lower frequencies than the corresponding mode of carboxylic groups of Asp or Glu, because of the influence of the heme macrocycle (20, 21). A frequency above 1728 cm^-1^ was proposed, however, for a protonated propionic group of heme b_D_ in dihemic quinol:fumarate reductase (22), but this high ν(C=O) mode frequency was rationalized by a hydrophobic environment of the propionic acid. In HZS, both propionic groups of heme γI are involved in hydrogen bonding interactions with water and/or amino acid side-chains (Fig. 2*A*) and a contribution at 1734 cm^-1^ is therefore not expected. We, thus, tentatively assigned the positive band at 1734 cm^-1^ to the redox-coupled protonation of an amino acid carboxylic group. According to the structural environment of heme γI, this group could be Asp168, located at 4.08 Å of the heme iron and in hydrogen bonding interaction with the water molecule axial ligand to the heme iron (Fig. 2*A*). Glu122, in hydrogen bonding interaction with one of the propionates of heme γI, could be another candidate for the absorbance changes at 1734 cm^−1^ and 1580 cm^−1^.

The spectrum in Figure 11*A* furthermore showed an intense difference band at 1609/1595 cm^-1^ and a positive band at 1393 cm^-1^ that could correspond to the ν_as_(COO^-^) and ν_s_(COO^-^) modes of a heme propionate group. Indeed, the propionate ν_as_(COO^−^) and ν_s_(COO^-^) modes have been reported at 1620–1540 cm^− 1^ and 1420–1360 cm^− 1^, respectively (21). The high intensity of the band at 1393 cm^-1^ probably points to a specific ionic interaction. For heme γI, one of the propionates forms an interaction with the side chain of Arg 143 (Fig. 2*A*) that may be altered upon the heme reduction. Such a change in interaction could also result in changes in the infra-red (IR) mode of the arginine side-chain. The ν_as_(guanidium) IR mode was reported at ≈1673 cm^-1^ in solution (18) and at higher frequencies (1680–1695 cm^−1^), upon interaction of the positively charged guanidium group with oxyanions (23). Further investigation, notably using samples in D_2_O, will allow testing this hypothesis. Unfortunately, our attempts to record FTIR difference spectra in this potential range in D_2_O failed so far due to the small signal size in this potential range and the instability of the sample after the H_2_O/D_2_O exchange.

Identification of heme skeletal modes was based on previous thorough investigations on protoporphyrin-(methyl)imidazole complexes, 5-coordinated high-spin (5c-HS) and 6-coordinated low-spin (6c-LS) microperoxidases, and *c*-type cytochromes (19, 24, 25). A broad signal of relatively low amplitude is present in the spectrum in Figure 11*A* at 1244-1229 cm^−1^. It most probably stems from the ν_42_ δ(CmH) skeletal heme modes of the reduced heme species and its broad shape and low amplitude is a characteristic of high-spin hemes.

This feature and signals in the 1150 cm^-1^ to 1120 cm^-1^ region clearly distinguish the spectrum of Figure 11*A* from the one in 11B. The latter was recorded in the −210 mV to −560 mV range and thus reflects vibrational changes associated to the redox transition of the two low potential hemes around −330 mV. Here, a negative band at 1126 cm^-1^ is attributed to the ν(Pyr Half ring) mode, previously assigned in oxidized LS 6c imidazole-microperoxidase complex (25) and reflects, together with the sharp band of relatively strong amplitude at 1241 cm^−1^, assigned to the ν_42_ δ(CmH) skeletal heme mode, the low spin character of the hemes that react in the low-potential range. Further positive bands at 1531 cm^−1^ and 1417 cm^−1^ in both spectra were assigned to the ν_38_ (CbCb) and ν_41_ (CaN) skeletal modes of reduced hemes, which are not sensitive to the spin state.

By splitting the −210 mV to −560 mV potential step in two separate ranges, from −210 mV to −360 mV (Fig. 11*C*) and from −410 mV to −610 mV (Fig. 11*D*), we observed significant differences in the spectra, notably in the 1700-1550 cm^-1^ region. Although we cannot completely separate the contributions from the two low-potential hemes, the FTIR spectra were consistent with the dominant contribution of distinct heme species in the two different low-potential windows and clearly showed that the two hemes have slightly different redox midpoint potentials. As proposed from optical spectroscopy, spectra in Figure 11, *C* and *D* should have dominating contributions from heme γII and heme αII, respectively.

A first difference between these two spectra concerned the IR signature of the axial histidine ligands in the oxidized state. The ν(C_5_-N_1_) ring mode of the histidine axial ligands of heme γII was identified as a strong band at 1099 cm^-1^ for the oxidized state in Figure 11*C*, while the corresponding band was more complex with a shoulder at 1108 cm^-1^ in spectrum 11D, containing dominating contributions from heme αII. The frequency of this band depends on the strength of the Fe-Histidine interaction, on the type of imidazole coordination to the iron, with Nτ or Nπ, and on the protonation state or electronegativity of the histidine imidazole ring (24, 25, 26, 27).

**Figure 12:**
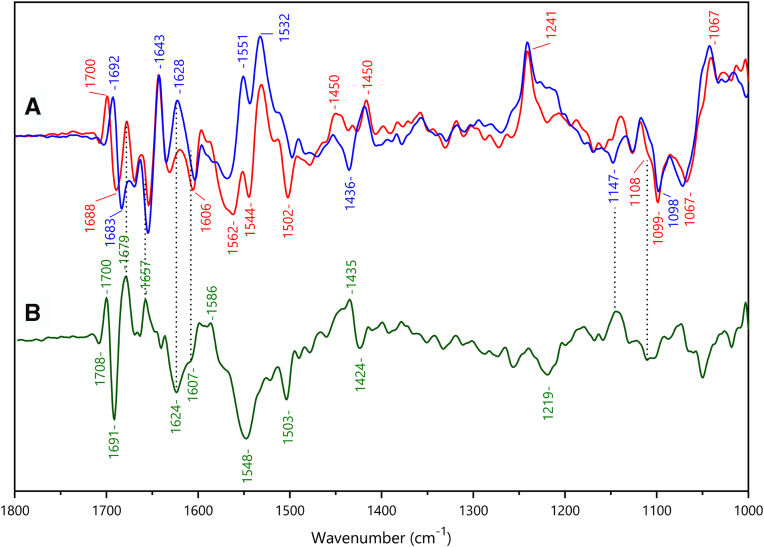
**Electrochemically induced FTIR difference spectra of *Kuenenia stuttgartiensis* HZS at -560 mV minus -210 mV.***A*, pH 8 (*red*) and pD 8 (*blue*). *B*, difference spektrum (*green*) pH 8 minus pD 8. HZS, hydrazine synthase.

The band at 1108 cm^-1^ is typical for a Nτ coordinated neutral histidine side chain, while the band at 1099 cm^-1^ could either correspond to a deprotonated histidine or highly electronegative Nτ coordinated neutral ligand of the heme iron (26, 27). We could exclude contribution from a Nπ coordinated neutral histidine side-chain, since spectra recorded in D_2_O (Fig. 12*A*, note that the spectra of Fig. 12*A* have been recorded applying potentials of −210 mV and −560 mV, allowing the redox reaction of the two hemes) did not show the expected upshift of 10 cm^-1^ of the corresponding imidazole vibration frequency, (27). Inspection of the crystal structure reveals that both histidine, His229 or His332, are Nτ coordinated to heme γII and have no possible hydrogen bonding partner that could stabilize an imidazolate suggesting that the low frequency results in strong His-Fe(III) interactions. However, the structure of HZS might be its reduced state, due to the reducing character of X-ray radiation, so that, in the oxidized state, structural rearrangement may stabilize one imidazolate ligand. Finally, the negative signal at 1108 cm^-1^, characteristic of the oxidized heme αII, is probably slightly downshifted in the spectrum recorded in D_2_O at pD 8 (Fig. 12 A, blue), which would confirm its attribution to the ν(C_5_-N_1_) ring mode of a Nτ coordinated His.

Prominent differences between the FTIR difference spectra in Figure 11, *C* and *D* were observed in the 1700-1600 cm^-1^-region, with strong negative bands at 1690 and 1632 cm^-1^ mainly visible in the spectrum with predominant heme αII contribution (Fig. 11*D*). This region is complex as peptide groups as well as side-chain modes from Arg, Lys, Asn or Gln may contribute (reviewed in Barth 2007 (28)). In contrast to peptide and heme modes, Arg, Asn, or Gln side-chain modes downshift by at least 30 cm^-1^ in D_2_O (reviewed in Barth 2000 (29). We thus analyzed the effect of H_2_O/D_2_O exchange in this spectral range (Fig. 12). The difference spectra recorded for the −210 mV to −560 mV potential step at pH 8 in H_2_O and D_2_O contain contributions from hemes αII and γII (Fig. 12). Inspection of the X-ray structure in the vicinity of heme αII reveals two arginine residues, Arg748 and Arg760, each at H-bonding distance to one propionate of heme αII, and a distance of 3.08 Å between Asn693 peptide carbonyl and the Nπ of the His689 heme ligand. No Arg, Lys, Asn, or Gln side chains are present in the close environment of heme γII, *i.e.* at distances less than 4 Å of heme γII or its His ligands.

A differential signal, positive at 1700 cm^-1^ and negative at 1688 cm^-1^ in the spectrum recorded in H_2_O (Fig. 12*A*, red) appears downshifted to 1692/1683 cm^-1^ in D_2_O (Fig. 12 A, blue). This signal is therefore proposed to account for a peptide carbonyl mode. This shift appears as a differential signal 1700/1691 cm^-1^ in the double difference spectrum (pH 8 *minus* pD 8, Fig. 1*B*). In this spectrum two positive bands at 1679 and 1657 cm^−1^ seem to have a broad negative counterpart at 1624 cm^−1^ with a shoulder at 1607 cm^−1^. A simple interpretation of these signals is that two positive bands at 1679 and 1657 cm^−1^ in the difference spectrum at pH 8 downshift by 55 and 50 cm^−1^ in D_2_O, respectively. According to literature data, such downshifts are best explained by contributions from arginine side chains. The ν_as_ and ν_s_ (C_2_N_3_H_5_) guanidinium modes, at 1675 to 1670 cm^−1^ and 1640 to 1630 cm^−1^ in H_2_O are downshifted by 65 and 47 cm^−1^ in D_2_O, respectively (29). Interaction of the guanidinium group with ions and hydrogen bonding upshifts the ν_as_(C_2_N_3_H_5_) mode (23, 30, 31). The bands at 1679 and 1657 cm^−1^ arise either from the ν_as_ and ν_s_ (C_2_N_3_H_5_) modes from one arginine or to the ν_as_(C_2_N_3_H_5_) modes of the two arginines (Arg748 and Arg760) interacting with the propionates of heme αII, which undergo an intensity increase rather than a frequency shift upon heme αII reduction.

Strong differences in the 1560 to 1540 cm^-1^ region of spectra in Figure 12*A* correspond to a large negative band at 1548 cm^-1^ in the double difference spectrum of Figure 12*B*, which is assigned to amide II modes. These modes are expected to downshift by 60 to 100 cm^-1^ upon deuteration, and the positive signature at ≈ 1440 cm^-1^ in Figure 12*B* can account for the amide II contribution in D_2_O. D_2_O insensitive bands at 1532(+) cm^-1^ are attributed to the ν_38_ (CbCb) heme mode ((25) and references therein).

## Discussion

As detailed above, the reaction mechanism of the unique enzyme HZS is not yet known. Hypothesis based on the structure and general chemistry knowledge involve reactions of nitrogen components on two of the four *c*-type hemes. Redox properties, coordination and ligand changes of these hemes are important parameters to support the currently proposed reaction mechanism or come up with a variant. Here, we performed optical, EPR, and FTIR spectroscopy on HZS, in order to provide an in-depth characterization of the individual HZS hemes and their protein environment, thereby unveiling unexpected properties of the two catalytic hemes. Heme γI, the candidate for NO reduction to hydroxylamine can adopt different ligation states and heme αI, proposed to catalyze the comproportionation of hydroxylamine with ammonium to form hydrazine cannot be reduced. The properties of all four HZS hemes are summarized in Table 1.

**Table 1:** Spectral characteristics, redox midpoint potentials, and amino acid residues affected by redox transitions of the different hemes from HZS

	Heme γII	Heme γI	Heme αII	Heme αI
Visible	Ox:	Ox:	Ox:	Ox:
	403 nm	403 nm	403.5 nm	398.5 nm
	Red:	CT: 620 nm	Red:	CT: 615 nm
	420, 553	Red:	420, 548, 554.5 nm	
		420,430, 554 nm	Split alpha band	
		Mixed HS/LS		
EPR	g = 3.46	g = 6	g = 3.08	g = 6.29/5.65
		g = 2.44/2.23/1.90		
		g = 2.50/2.27/1.86		
		g = 2.57/2.21/1.80		
		Fe-NO as 5th ligand g ≈ 2 (ΔG = 17)		
*E*_*m*_*(versus SHE)*	∼ −310 mV	∼ −30 mV	∼ −360 mV	Irreducible
FTIR	Fe-His, low-spin heme ν_42_ δ(CmH) mode	Low-/high-spin heme ν_42_ δ(CmH) mode, COO^-^(ox)/COOH(red) of Asp168/Glu122	Fe-His low-spin heme ν_42_ δ(CmH) mode Arg – propionate COO^-^ interaction	-

CT, charge transfer.

Low spin heme αII had the lowest redox midpoint potential of all hemes in the enzyme and exhibited a split alpha band. High-spin heme αI could not be reduced and exhibited a rhombic EPR signal. This heme was proposed to catalyze the comproportionation reaction of hydroxylamine with ammonium. No net electron exchange is involved in this reaction. Su and Chen (32), however, proposed from density functional theory (DFT) calculations that the reduced state of the heme would be the lowest energy state in an NH_2_OH-bound intermediate state and should therefore be the active redox state. Our data did not confirm this hypothesis since heme αI remained oxidized in all tested conditions. Addition of substrate to the sample (data not shown) did not change this. We are also confident that the observed signal is not due to an artefact of our purification procedure since it was already present in the crude cell extract and there, too, did not react with reductants. A choice of atoms included in the calculations of Su and Chen (32), probably too restrictive, and an omission to calculate the energies for states M2 and M3 for the oxidized heme may be part of an explanation for these discrepancies. The data presented here may encourage a new round of calculations, with an oxidized heme αI as starting point.

Low-spin heme γII showed a redox midpoint potential slightly higher than heme αII, whereas high-spin heme γI adopted multiple spin and redox states. The observed redox transitions of γI were higher than the ones for the low spin hemes and occurred around 0 mV. As shown previously, this heme γI was fully reduced upon addition of reduced TH, the putative electron donor of HZS with redox potentials of −400 mV and −190 mV, whereas the HZS HALS signals were only partially reduced (13). Furthermore, in its reduced state, part of the heme γI population bound NO.

The γ-subunit is homologous to bacterial di-heme cytochrome *c* peroxidases (bCcP) and MauG proteins, sharing a similar protein fold and structural arrangement of the hemes. The HZS γ-subunit indeed showed certain spectral characteristics reminiscent of these proteins, but also some differences. In bCcP, one of the hemes is low spin with a highly anisotropic EPR signal, the other one high spin with an axial g = 6 signal. In MauG, both hemes are low spin with exception of a very small high spin population. In HZS, heme γII has a HALS signal, as it is the case in bCcP. Heme γI, however, could adopt different spin states as reflected by the EPR and visible signals. Part of the population exhibited an axial EPR signal at g = 6, like bCcP (33, 34) and the minor high-spin population in MauG (35). An additional part of the population of heme γI exhibited low spin characteristics, observed as a main contribution in MauG, giving rise to one to three signals in the range of g_z,y,x_ at 2.44, 2.23, 1.90, at 2.50, 2.27, 1.86 and at 2.57, 2.21, 1.80. This mixture of spin states may explain why the IR signature of the reduced heme γI ν_42_ δ(CmH) mode significantly differs from that of a (bis-histidine) low spin heme. We do not yet have proof for the sixth ligand that could confer these spectral characteristics to heme γI, but a hydroxyl ion as proposed for bCcP (34) would be a plausible hypothesis in line with the EPR signature (36). This was supported by the HZS structure, where a water molecule was detected in hydrogen bonding distance to the heme iron. The fact that we detected spectral characteristics for a six coordination of heme γI in EPR spectra and in optical spectra precluded that they are due to freezing artefacts as suspected for bCcP.

MauG and HZS both are supposed to catalyze redox reactions involving several electrons at their active site. Although for MauG strong redox cooperativity was reported for the two hemes, making them behave as a redox entity, no such observation could be made for HZS. Indeed, for MauG the redox cooperativity translated during a redox titration to a two-step reduction with identical spectral characteristics for both steps (37, 38). This was clearly not the case for HZS where not a two- but a three-electron transition was proposed to occur at the active site during the reduction of NO to hydroxylamine. The presently determined redox midpoint potentials of the hemes in the γ-subunit would allow for reduction of NO to hydroxylamine, which occurs at an overall redox potential of −30 mV. FTIR further provided evidence for Asp168 protonation upon heme reduction, which suggests a direct role in proton supply for hydroxylamine formation at heme γI. We do not know whether catalysis involves several steps and where the three electrons necessary to complete the reaction stem from. Oxidation of an aromatic amino acid side chain, as observed for KatG (39), a member of the MauG family, might be an option that will be investigated in the future. Irreversible binding of NO to a reduced heme has to be avoided. Further experiments are necessary to investigate whether NO bound as a fifth ligand to a heme, as observed in this work, is an on-pathway intermediate state, as reported for soluble guanylate cyclase (40) or constitutes a stable off-pathway species, as seen in cytochrome P460 (41). Appearance of the signal at −210 mV and subsequent disappearance below −360 mV during electrochemically poised EPR spectra (Fig. 7) may be a hint in favor of the first hypothesis. However, further experiments are needed to explore the reaction mechanism with NO.

Another interesting observation concerned the kinetics of heme reduction: heme αII was rapidly reduced by dithionite as expected for a surface exposed heme (Fig. 3, red line). Heme γII, which is exposed to the surface of the protein as well, however, needed minutes to get reduced by dithionite as did heme γI. The sluggish reduction behavior of the hemes in the γ-subunit, despite their more positive equilibrium redox midpoint potentials, when compared to heme αII, hints to structural and/or ligand environment changes linked to the redox transition. This was strengthened by the variability in spectroscopic signatures and redox midpoint potential observed for heme γI over different redox cycles, at different pH values and between different experiments. Further FTIR experiments in the presence of substrates and in D_2_O might help understanding the protein dynamics surrounding heme γI.

In conclusion, we could identify and attribute distinct spectral properties to the four different HZS hemes and determine their redox properties. The reduction potentials of hemes γII & γI, and the ability of heme γI to bind NO are in line with the proposed reduction of NO to hydroxylamine by the HZS γ-subunit. Heme αI could not be reduced, indicating the second half reaction likely starts on an oxidized heme. Identification of unique EPR and optical spectral features of this heme can assist in determining its reaction mechanism by observing changes under substrate addition. All in all, our data provide the basis to further unravel the reaction mechanism of this unique enzyme.

## Experimental procedures

### Protein purification

HZS purification was adapted from Kartal *et al.*, 2011 (3) with some adjustments. All steps of the HZS purification procedure were performed anaerobically. Subsequently, 1.5 L of anaerobic *K. stuttgartiensis* MBR1 bleed (absorbance ∼ 1.2) (42) was centrifuged at 5000 g, 15 min, 4 °C in an Allegra X-15R centrifuge (Swinging bucket rotor, Beckman Coulter). The cell pellet was resuspended in 10 ml 20 mM Tris pH 7.5 and passed once through a French press at 120 MPa. The resulting crude extract was subjected to ultracentrifugation at 162,000g, 50 min, 4 °C (Fixed angle Ti90 Rotor, Optima XE90, Beckman Coulter). The supernatant was loaded onto a 70 ml Q-sepharose column (XK 26/20, GE Healthcare/Cytiva) equilibrated with 20 mM Tris pH 7.5, connected to an Äkta purifier (GE Healthcare/Cytiva) and run at a flow rate of 5 ml/min. HZS was eluted by a step to 20 mM Tris, 200 mM NaCl pH 7.5 and concentrated on a 50 kDa cutoff Amicon pressure filter unit. After buffer exchange to 20 mM potassium phosphate, 100 mM NaCl pH 7, the sample was applied to a 10 ml ceramic hydroxyapatite Type-II 40 μm (Bio-Rad) column (Omnifit, Diba Industries) equilibrated in the same buffer and run at 5 ml/min. First a step of 46 mM potassium phosphate, 100 mM NaCl pH 7 was applied to remove contaminants, after which HZS was eluted with a step of 98 mM potassium phosphate, 100 mM NaCl pH 7. HZS was concentrated on a 100 kDa Vivaspin spin filter (Sartorius) and stored at −20 °C in anaerobic serum bottles until use. HZS purity was checked using gel electrophoresis (43) and its identity confirmed using MALDI-ToF MS.

HZS α-subunit was purified by applying the 200 mM NaCl fraction from the Q-sepharose column on a 50 ml hydroxyapatite type II (Bio-Rad) column (XK 26/20, GE Healthcare/Cytiva) equilibrated with 20 mM potassium phosphate pH 7 at 5 ml/min. A step to 20 mM potassium phosphate, 1 M NaCl was applied and the eluted sample concentrated and buffer exchanged to 20 mM potassium phosphate pH 7, before it was reapplied on the 30 ml hydroxyapatite column. A linear gradient of 0 to 1 M NaCl was applied and HZS α-subunit eluted at a conductivity of 65 mS/cm. A 100 kDa cutoff spin filter (Sartorius) was used to concentrate the sample and purity was assessed by gel electrophoresis.

Upon initial purification by Kartal *et al.*, 2011 (3), HZS was identified as the gene product of ORFs kuste2861 (*hzsA*), kuste2859 (*hzsB*), and kuste2860 (*hzsC*), based on the genome sequenced by Strous *et al.* in 2006 (44). Resequencing of the complete *K. stuttgartiensis* genome by Frank *et al.* in 2018 (42), revealed that a novel strain had become dominant in the bioreactor, which contained two complete HZS gene clusters (*hzsABC*, KSMBR1_3603-KSMBR1_3601 and KSMBR1_2713-KSMBR1_2711) and a third copy of *hzsB* (KSMBR1_2703) *and hzsC* (KSMBR1_2704). After removal of signal peptides, the different copies of the HZS subunits are 100% identical on an amino acid level, also to those of the initial strain.

### MALDI-ToF MS

Maldi-ToF MS was carried out according to Farhoud *et al.*, 2005 (45). Briefly, bands of interest were excised from an SDS-PAGE gel and destained. After reduction and alkylation of cysteines with iodoacetamide, proteins were tryptically digested overnight. Peptides were extracted in 0.1% trifluoroacetic acid in 50% acetonitrile, mixed in a 1:1 ratio with a 10 mg/ml 4-hydroxy-a-cyanocinnamic acid matrix solution and spotted on an MSP 96 stainless steel plate (Bruker). MALDI-ToF MS analysis was performed on a Microflex LT (Bruker), and peptides were mapped against an in-house *K. stuttgartiensis* MBR1 database using BioTools software, with carbamidomethylation as global and methionine oxidation as a variable modification, allowing for one missed cleavage site and a 0.3 Da mass tolerance.

### UV-visible spectroscopy

UV-visible spectra were recorded aerobically on a Cary 60 spectrophotometer (Agilent) in a quartz cuvette (Hellma). After recording of ‘as isolated' spectra, samples were reduced using sodium dithionite. Origin 2020 (OriginLab Corp) was used to analyze the spectra.

### Optical redox titration

Optical redox titrations were performed aerobically using an optically transparent thin-layer electrochemical cell (46) with CaF_2_ windows and a path length of less than 10 μm or a plexiglas version of similar design with an optical path length of about 100 μm. Gold grid (Buckbee-Mears) working electrodes were surface-modified by boiling for 10 min in a 1 mg/ml PATS-3 solution. A platinum wire was used as a counter electrode and an Ag/AgCl wire in a 3 M KCl solution was used as a reference electrode, which was calibrated for SHE by titration of horse heart cytochrome *c*. The optically transparent thin-layer electrochemical cell was connected to a potentiostat and spectra were recorded on a customized Cary14 spectrophotometer from 700 to 375 nm at 10 °C. The sample consisted of ∼1 mM HZS, 100 mM Tris buffer pH 8, 50 mM KCl, and 40 μM (or 20 μM in the Plexiglas cell) mediators each (ferricyanide (E^0^′ = +430 mV); 1,4-benzoquinone (E^0^′ = +280 mV); 2,5-dimethyl-1,4-benzoquinone (E^0^′ = +180 mV); 1,2-naphthoquinone (E^0^′ = +145 mV); phenazine methosulfate (E^0^′ = +80 mV); 1,4-naphthoquinone (E^0^′ = +60 mV); phenazine ethosulfate (E^0^′ = +55 mV); 5-hydroxy-1,4-naphthoquinone (E^0^′ = +30 mV); 1,2-dimethyl-1,4-naphthoquinone (E^0^′ = 0 mV); 2,5-dihydroxy-p-benzoquinone (E^0^′ = −60 mV); 5,8-dihydroxy-1,4-naphthoquinone (E^0^′ = −145 mV), 9,10-anthraquinone (E^0^′ = −184 mV), 9,10-anthraquinone-2-sulfonate (E^0^′ = −225 mV); benzyl viologen (E^0^′ = −350 mV); and methyl viologen (E^0^′ = −440 mV/−772 mV).

Titrations were performed between +390 and −610 mV *versus* SHE in both reductive and oxidative directions in 30 mV steps, with a 15 mV difference between reductive and oxidative cycles and 10 min equilibration time for each step. The spectra were analyzed by global fitting using the mfit-nernst function of the QSOAS software (47) to extract redox potentials and the corresponding difference spectra and plotted using Origin 2020. In addition, specific wavelengths were investigated as a function of potential (*i.e.* 406 nm minus 412 nm) using Origin 2020 and potentials were extracted by fitting to a sum of Nernst curves. The error in the obtained midpoint potentials was estimated at ± 20 mV. Overall integrity of the HZS sample was verified by comparing the raw spectra at fully oxidized and fully reduced conditions over multiple cycles, which indicated that not more than 5% of the sample intensity was lost.

### EPR spectroscopy

HZS samples were prepared in an anaerobic glovebox in 100 mM Mops pH 7 and loaded into quartz EPR tubes, sealed with butyl rubber stoppers and frozen inside the glovebox in a cold finger. Spectra were recorded on an Elexsys E500 X-band EPR spectrometer at 9.48 GHz fitted with a helium cryostat (ESR900, Oxford instruments) for temperature control and a rectangular standard cavity (ER4102ST, Bruker). HZS was reduced by addition of one equivalent of sodium dithionite per HZS heme and reoxidized by addition of one equivalent of ferricyanide per HZS heme. Parameters of the spectra are given in the figure legends. For experiments with nitric oxide, 45 μM HZS was incubated in capped serum bottles containing a 5% NO headspace (95 μM in solution) in the glove box.

### Electrochemical preparation of EPR samples

2 ml of 50 μM HZS with the same mediator mix as used for optical titrations at 20 μM in Mops/KCl buffer pH 7 was deposited on a carbon felt. A reference and a counter electrode were connected *via* a salt bridge to the sample. The set-up was localized inside a glove box, potential was applied for several minutes before 200 μl of the sample was transferred to an EPR tube, capped, and frozen in a cold finger inside the glove box.

### Electrochemically induced Fourier transformed infrared difference spectroscopy

Sample preparation for FTIR experiments was identical to that for optical redox titrations. Exchange from H_2_O for D_2_O was performed in a glove box. In addition, 14 μl of approximately 1 mM HZS with mediators was diluted in 40 μl D_2_O, incubated for 30 min before water was blown off by a stream of nitrogen. The sample was diluted once more by 250 μl D_2_O and incubated overnight at 4 °C before being concentrated under a nitrogen stream. Before the sample was installed in the FTIR spectrophotometer, integrity was verified by taking a few optical spectra at different potentials. Then the cell was mounted in a Bruker Tensor 27 spectrometer, connected to a cooling system set at 10 °C and left for 2 h to remove water vapor in the spectrometer. Difference spectra were recorded in a high potential range (+390 to −210 mV) and low potential range (−210 to −560 mV). At each potential the sample was equilibrated for 10 to 15 min before 300 scans were acquired and averaged. Several cycles were collected (>20) for each potential step and individual difference spectra were manually assessed before data from each cycle was averaged using the OPUS 7.5 software. Baseline drifts and sample loss were corrected for by subtracting the oxidized minus reduced from the reduced minus oxidized spectra.

### Structural analysis

HZS structural data (PDB: 5c2v) were analyzed using Pymol software (Schrödinger, LLC).

## Data availability

Most data described within this manuscript are shown. EPR spectra recorded upon exposure of the anaerobically purified enzyme to oxygen or to hydrazine can be obtained on request from the corresponding author (baymann@imm.cnrs.fr).

## Conflict of interest

The authors declare that they have no conflicts of interest with the contents of this article.
